# Urgent Mitral Commissurotomy During Labor in Critical Rheumatic Mitral Stenosis

**DOI:** 10.7759/cureus.115113

**Published:** 2026-08-24

**Authors:** Mehdi Guedira, Hind Hibatouallah, Aida Soufiani, Nesma Bendagha, Nadia Fellat

**Affiliations:** 1 Cardiology Department, Ibn Sina Hospital, Mohammed V University, Rabat, MAR

**Keywords:** inoue balloon, labor, mitral stenosis, percutaneous mitral commissurotomy, pregnancy, rheumatic heart disease

## Abstract

A 32-year-old woman with critical rheumatic mitral stenosis presented at 39 weeks of gestation in early labor with ruptured membranes, having missed a percutaneous mitral commissurotomy planned for the second trimester. She was hemodynamically stable, with clear lungs and an oxygen saturation of 95% on room air, but reported dyspnea at rest corresponding to New York Heart Association class IV despite uninterrupted medical therapy. Echocardiography showed a mitral valve area of 0.55 cm², a mean transmitral gradient of 17 mm Hg, pulmonary artery systolic pressure of 58 mm Hg, a Wilkins score of 9 driven mainly by subvalvular thickening, and dense left atrial spontaneous echo contrast without thrombus. With the cervix dilated to 2 cm, a multidisciplinary team performed urgent Inoue-balloon commissurotomy two hours after admission. Mitral valve area increased to 1.60 cm², the mean gradient decreased to 6 mm Hg, the mean left atrial pressure fell from 37 to 26 mm Hg, and mitral regurgitation remained minimal. Spontaneous vaginal delivery of a term newborn followed approximately nine hours later, with Apgar scores of 8 at one minute and 10 at five minutes. No immediate maternal, obstetric, neonatal, or procedural complication occurred. This case illustrates individualized multidisciplinary sequencing of valve intervention and delivery when critical mitral stenosis is encountered after labor has already begun.

## Introduction

Rheumatic mitral stenosis remains an important cause of cardiovascular morbidity during pregnancy, particularly in regions where rheumatic heart disease is prevalent [1,2]. The increase in circulating volume, heart rate, and cardiac output may transform a previously tolerated fixed mitral obstruction into pulmonary venous hypertension and acute heart failure [3,4]. Management is especially challenging when a patient with critical stenosis presents at term after labor has begun, because the risks of progressive labor, the immediate postpartum volume shift, catheter intervention, and urgent operative delivery must be weighed within a narrow therapeutic window [1,2].

Percutaneous mitral commissurotomy, in which a balloon-tipped catheter is advanced across the interatrial septum and inflated to split the fused valve commissures, is well established during pregnancy. Current guidelines and the available evidence base, however, address elective intervention undertaken antenatally, predominantly in the second or third trimester and before the onset of labor [1,2,5]. No recommendation specifies how valve intervention and delivery should be sequenced once a patient with an untreated critical obstruction presents in established labor. This gap leaves the treating team to weigh an unproven catheter procedure in an actively laboring patient against delivery with the obstruction unrelieved.

We report the successful use of urgent percutaneous mitral commissurotomy performed during early labor, approximately nine hours before spontaneous vaginal delivery, in a patient whose planned second-trimester procedure had not been performed.

This case was previously presented as an unpublished poster at the 2025 Journées Scientifiques de l'Internat, held at Rabat University Hospital Center. The clinical information reported in this manuscript was obtained from the original medical records.

## Case presentation

History of presentation

A 32-year-old woman, gravida 2 para 1, with blindness presented at 13 weeks of gestation with New York Heart Association functional class II dyspnea. Transthoracic echocardiography demonstrated severe rheumatic mitral stenosis, with a mitral valve area of 0.50 cm² and an estimated pulmonary artery systolic pressure of 61 mm Hg before any treatment. Beta-blocker and diuretic therapy were prescribed, and percutaneous mitral commissurotomy was planned for approximately 22 weeks of gestation. The patient did not attend the scheduled appointment and was subsequently lost to follow-up; she consulted no physician during the intervening interval but continued her prescribed medication. She next presented at 39 weeks of gestation, at 09:00, in labor with ruptured membranes. She reported several hours of contractions before arrival, although the exact duration of labor before admission could not be established from the available records.

Physical examination

At admission, blood pressure was 120/70 mm Hg and heart rate was regular at 68 beats/min. Oxygen saturation was 95% on room air, and no supplemental oxygen was required. Pulmonary auscultation was clear, and there were no clinical signs of systemic congestion. She nevertheless reported dyspnea at rest, persisting between contractions and corresponding to New York Heart Association functional class IV, having deteriorated from class II earlier in pregnancy despite uninterrupted beta-blocker and diuretic therapy. Cardiac auscultation revealed a diastolic murmur consistent with mitral stenosis. Uterine contractions occurred approximately every 10 minutes; cervical dilation was 2 cm, and the fetus was in cephalic presentation. Hemoglobin and other admission laboratory values were not retrievable from the available records.

Past medical history

Her medical history comprised rheumatic mitral stenosis and blindness. She had one previous pregnancy, which was uneventful. There was no other medical, surgical, or obstetric history, and she was taking no medication other than the beta-blocker and diuretic prescribed earlier in the pregnancy.

Differential diagnosis

In a term pregnancy, dyspnea may reflect normal physiological adaptation, pulmonary congestion caused by mitral stenosis, pulmonary embolism, infection, anemia, or cardiomyopathy. In this patient, the known critical valve obstruction and urgent echocardiographic findings strongly supported mitral stenosis as the principal cause of the symptoms. The immediate clinical problem was therefore not diagnostic uncertainty but selection and sequencing of valve intervention and delivery.

Investigations

**Figure 1:**
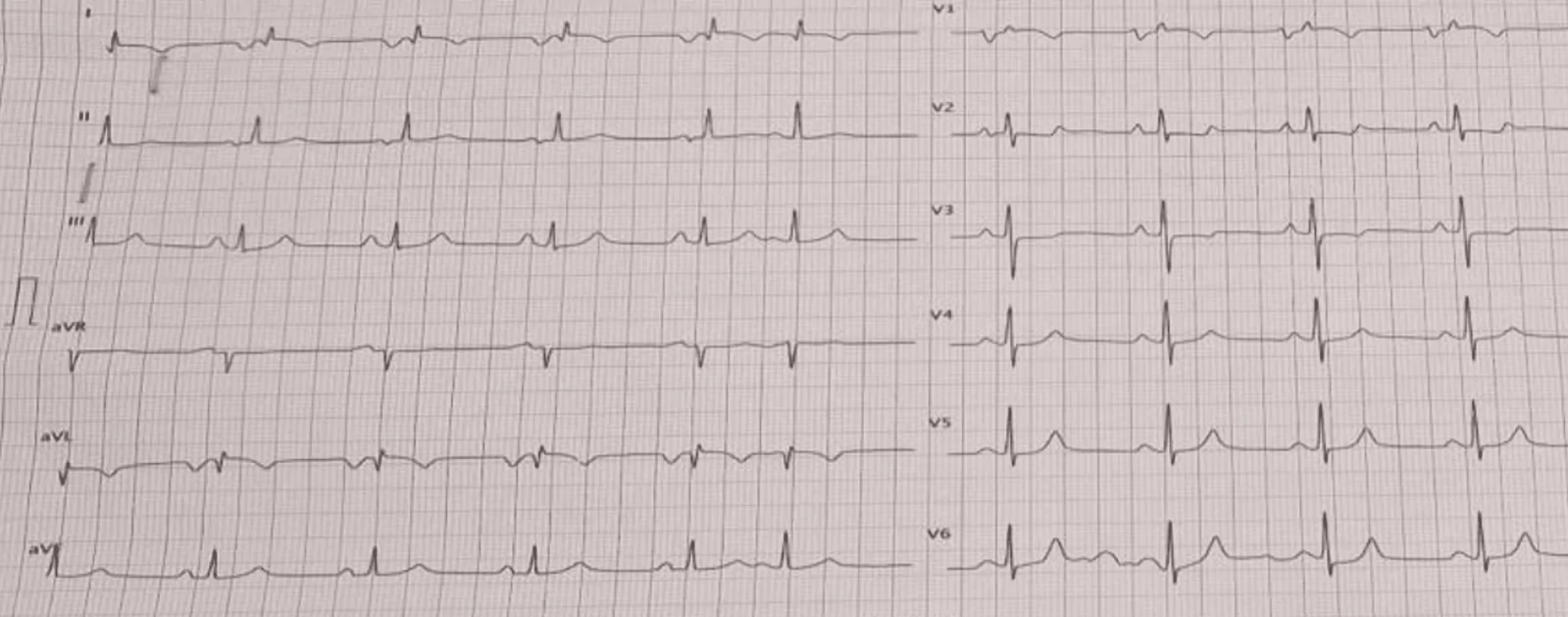
ECG at admission. Sinus rhythm at 65 beats/min, normal frontal axis, PR interval of 160 ms, left atrial abnormality, one premature atrial complex, and T-wave inversion in the high lateral leads.

The electrocardiogram showed sinus rhythm at 65 beats/min, a normal frontal axis, a PR interval of 160 ms, left atrial abnormality, one premature atrial complex, and T-wave inversion in the high lateral leads (Figure 1).

**Figure 2:**
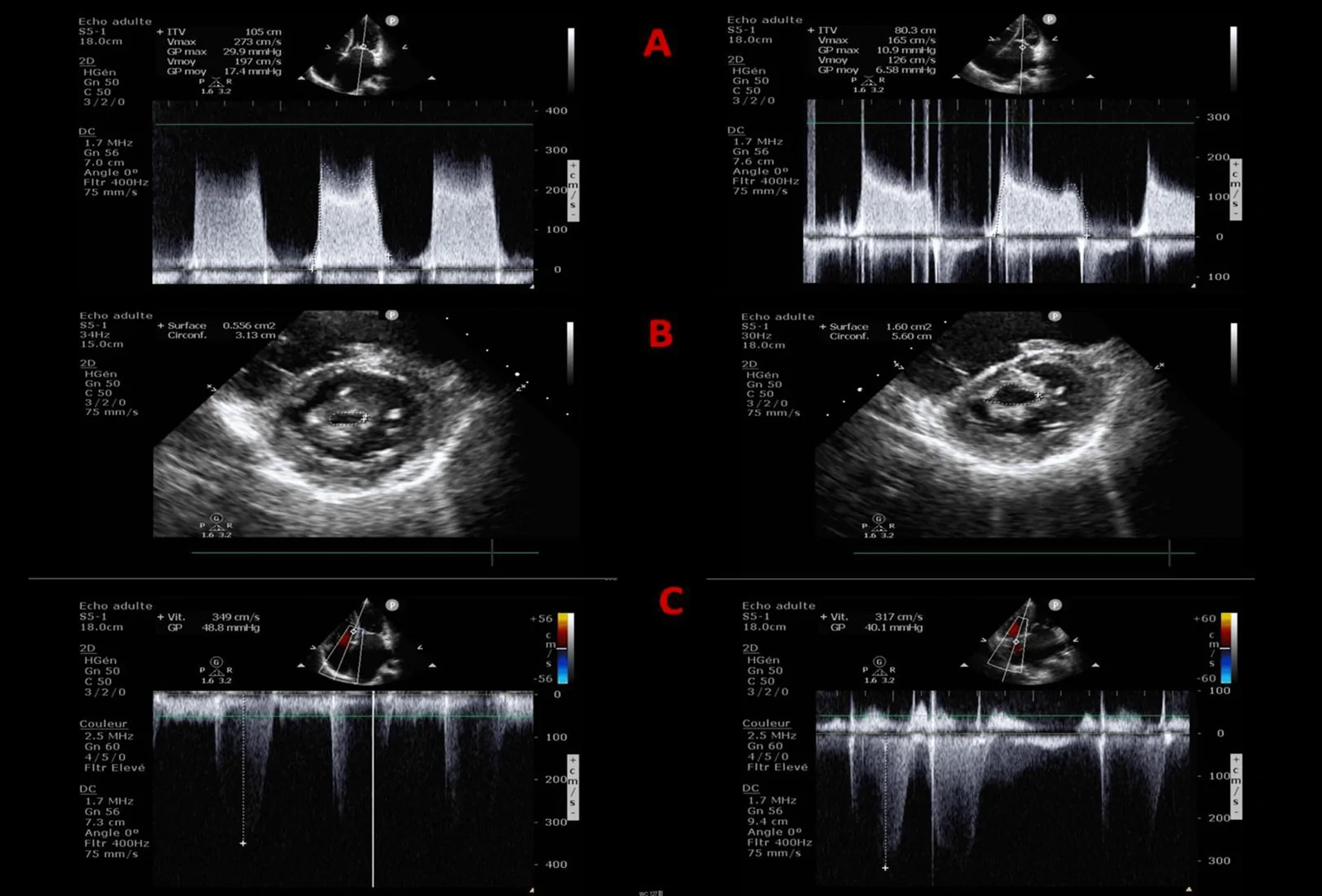
Transthoracic echocardiography before and after percutaneous mitral commissurotomy Left panels: baseline study. Right panels: study repeated immediately after the procedure. (A) Continuous-wave Doppler across the mitral valve, showing a mean transmitral gradient of 17.4 mm Hg at baseline and 6.6 mm Hg after commissurotomy. (B) Parasternal short-axis planimetry of the mitral orifice, showing a valve area of 0.55 cm² at baseline and 1.60 cm² after commissurotomy. (C) Continuous-wave Doppler of the tricuspid regurgitation jet, showing a peak gradient of 48.8 mm Hg at baseline and 40.1 mm Hg after commissurotomy.

Urgent transthoracic echocardiography supplemented by transesophageal echocardiography was performed at admission and showed critical rheumatic mitral stenosis. The mitral valve area was 0.55 cm² by planimetry, the mean transmitral gradient was 17 mm Hg, and the estimated pulmonary artery systolic pressure was 58 mm Hg, derived from a tricuspid regurgitation peak gradient of 48.8 mm Hg (Figure 2). Compared with the first-trimester study performed before treatment, the valve area was essentially unchanged, the small difference reflecting interstudy measurement variability, whereas pulmonary artery systolic pressure had fallen slightly from 61 mm Hg under beta-blocker and diuretic therapy. The left atrium was markedly dilated with dense spontaneous echo contrast, but transesophageal imaging excluded left atrial or appendage thrombus. Mitral regurgitation was trivial at baseline. The Wilkins score was 9, comprising two points each for leaflet mobility, leaflet thickening, and calcification, and three points for subvalvular thickening. The score was therefore driven predominantly by subvalvular disease rather than by leaflet calcification or restricted mobility, and the valve was considered suitable for a percutaneous approach despite the borderline total score.

Management

The case was discussed urgently by a multidisciplinary team comprising interventional cardiologists, an anesthesiologist-resuscitator, and an obstetrician-gynecologist. Although blood pressure and heart rate were preserved, class IV dyspnea, a critical valve area, pulmonary hypertension, and the anticipated rise in cardiac output during labor and immediately after delivery were considered to confer a substantial risk of pulmonary edema. The cervix was only 2 cm dilated, and contractions remained spaced, providing a limited opportunity to relieve the obstruction before the hemodynamically more demanding phases of labor. The team therefore selected urgent percutaneous mitral commissurotomy before transfer to the delivery room, rather than proceeding directly to delivery with an untreated critical obstruction or performing unscheduled cardiac surgery.

**Figure 3:**
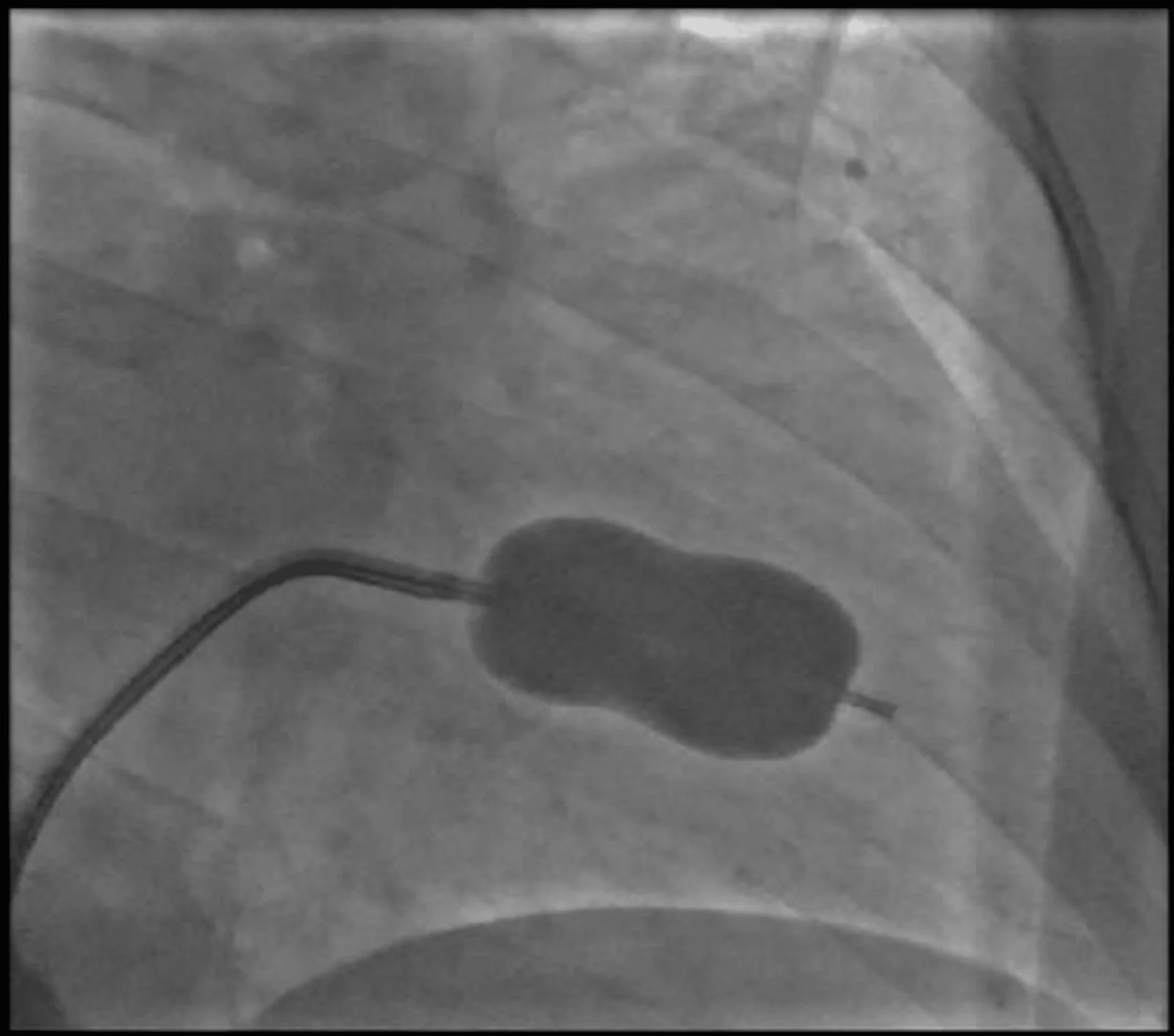
Balloon inflation across the mitral valve during percutaneous mitral commissurotomy. Fluoroscopic image obtained during percutaneous mitral commissurotomy showing inflation of the balloon catheter across the stenotic mitral valve to relieve the valvular obstruction.

Commissurotomy was performed at approximately 11:00, about two hours after admission. Continuous maternal cardiorespiratory monitoring and fetal cardiotocographic monitoring were maintained throughout the procedure; the fetal heart rate tracing remained normal, with no decelerations, including during balloon inflations. A 6-F pigtail catheter was positioned at the aortic root. Right femoral venous access was used, and transseptal puncture was performed with a Brockenbrough needle. Unfractionated heparin was administered intravenously after transseptal puncture at a minimum dose of 70 IU/kg, with subsequent adjustment guided by the activated clotting time. A target of 250 to 300 seconds was adopted, deliberately kept toward the lower limit given the anticipated delivery later the same day; the activated clotting time did not exceed 280 seconds at any point. Commissurotomy was performed with the Inoue technique. A 26-mm Inoue balloon was selected according to the patient's height (1.62 m; body weight 64 kg). Stepwise inflations were performed from 18 to 22 mm, each increment repeated twice, until disappearance of the mitral waist in a 30° right anterior oblique projection (Figure 3). Total fluoroscopy time was 13 minutes. No external abdominal lead shielding was used: at this stage of pregnancy the gravid uterus lies outside the direct beam, so fetal exposure results almost entirely from internal scatter, which external shielding does not attenuate, and shielding entering the field may trigger automatic dose-rate escalation and paradoxically increase exposure. The total radiation dose was not recorded in the available documentation. Left atrial pressure was recorded before and after dilation.

The procedure produced an immediate hemodynamic and echocardiographic improvement. The mitral valve area increased from 0.55 to 1.60 cm², and the mean transmitral gradient decreased from 17 to approximately 6 mm Hg (Figure 2). The mean left atrial pressure decreased from 37 to 26 mm Hg. The tricuspid regurgitation peak gradient decreased from 48.8 to 40.1 mm Hg. Post-procedural mitral regurgitation was minimal, and no procedural complication occurred. The patient remained clinically stable, with no supplemental oxygen requirement, and reported a slight subjective improvement in dyspnea, although the short interval before delivery precluded meaningful reassessment of functional class.

Vaginal delivery was then pursued. In the context of procedural anticoagulation, the anesthetic team selected multimodal intravenous analgesia rather than neuraxial analgesia. The patient had a spontaneous vaginal delivery of a term newborn approximately nine hours after commissurotomy, at approximately 20:00 on the same day, without instrumental assistance and with good maternal tolerance. Apgar scores were 8 at one minute and 10 at five minutes. There was no maternal, obstetric, neonatal, or procedural complication. Estimated blood loss and birth weight were not recorded in the available documentation.

Because the first hours after delivery carry the highest risk of pulmonary congestion in mitral stenosis, the patient was transferred to the coronary care unit for 24 hours of continuous noninvasive monitoring. Transthoracic echocardiography repeated on the first postpartum day showed a mitral valve area of approximately 1.60 cm² and a mean transmitral gradient of approximately 6 mm Hg, unchanged from the post-procedural study, with mitral regurgitation that remained minimal. The tricuspid regurgitation peak gradient had fallen further to 36 mm Hg, corresponding to an estimated pulmonary artery systolic pressure of 46 mm Hg with an estimated right atrial pressure of 10 mm Hg. The patient had not been receiving anticoagulation before admission; in sinus rhythm, with no left atrial thrombus and no previous embolic event, dense spontaneous echo contrast alone was not considered a formal indication for anticoagulation during pregnancy. Given the dense spontaneous echo contrast and the markedly dilated left atrium, low-molecular-weight heparin was started on the first postpartum day, a regimen compatible with breastfeeding, with subsequent transition to warfarin; the indication is to be reassessed at cardiology follow-up according to the persistence of thromboembolic risk factors. She was then transferred to a conventional ward and discharged on the third day.

## Discussion

Mitral stenosis is particularly sensitive to the physiological changes of pregnancy because transmitral flow is constrained by a fixed orifice. Plasma volume and cardiac output rise early in gestation, while pregnancy-related tachycardia shortens diastolic filling time. These changes increase the transmitral gradient and left atrial pressure and may lead to pulmonary edema, atrial arrhythmia, or right-sided pressure overload [1-3]. During labor, pain, anxiety, contractions, and autotransfusion further increase cardiac output. The immediate postpartum period remains hazardous because relief of aortocaval compression and uterine contraction abruptly return blood to the central circulation [1,4,6,7].

Current guidelines emphasize pre-pregnancy treatment of severe mitral stenosis whenever possible and multidisciplinary Pregnancy Heart Team care for high-risk patients. In a pregnant patient who remains markedly symptomatic or develops severe pulmonary hypertension despite medical therapy, percutaneous mitral commissurotomy is preferred to surgery when valve morphology is suitable [1,2]. Published series demonstrate high procedural success and substantial improvement in valve area, transmitral gradient, symptoms, and left atrial pressure, although fetal growth restriction, preterm birth, and low birth weight remain important in high-risk populations [8-10]. The present patient met these criteria: she had deteriorated to New York Heart Association class IV with a pulmonary artery systolic pressure of 58 mm Hg despite uninterrupted medical therapy, and her valve morphology remained amenable to a percutaneous approach.

The distinctive feature of this case is the timing of intervention after the onset of labor. A systematic review and meta-analysis of percutaneous balloon mitral valvotomy in pregnancy reported high procedural success with favorable maternal and fetal outcomes, but the procedures analyzed were undertaken antenatally, predominantly during the second and third trimesters [5]. A prospective series designed specifically to identify the optimal timing of intervention likewise compared strategies across trimesters rather than during labor [10]. We identified no comparative data on commissurotomy performed after labor has begun, and the published experience appears limited to isolated reports of intervention for symptomatic severe rheumatic mitral stenosis during pregnancy [11,12]. The present case therefore adds the practical dilemma of whether to intervene or to deliver when a patient with an untreated critical obstruction arrives at term with ruptured membranes and early cervical dilation.

Four strategies were weighed. Continuing labor under medical management alone would have left a valve area of 0.55 cm² unrelieved through the phases of greatest hemodynamic demand, in a patient already in class IV. Proceeding directly to cesarean delivery would have removed the labor-related rise in cardiac output but would have added the volume shifts, anesthesia, and bleeding risk of major abdominal surgery in a patient with fixed obstruction and pulmonary hypertension, without relieving the obstruction before postpartum autotransfusion; current guidance also favors vaginal delivery in most women with cardiovascular disease [1]. Deferring valve intervention until after delivery would have exposed the patient untreated to the very period of highest risk. Emergency surgical commissurotomy would have required cardiopulmonary bypass at term, with its attendant fetal risk, and could not have been organized within the available window. Urgent percutaneous commissurotomy was therefore preferred, because cervical dilation of only 2 cm and spaced contractions afforded a window in which the obstruction could be relieved before labor advanced.

Dense spontaneous echo contrast in a markedly dilated left atrium reflects low-velocity blood stasis and identifies a prothrombotic state even in sinus rhythm and in the absence of visible thrombus. Its significance is heightened in the peripartum period, when the physiological hypercoagulability of late pregnancy persists for several weeks. Transesophageal echocardiography excluding left atrial and appendage thrombus was accordingly a prerequisite before transseptal puncture. Anticoagulation was deferred until after delivery to avoid compounding peripartum bleeding risk, then started on the first postpartum day once hemostasis was secured.

The two procedural risks specific to this setting were mitigated rather than merely accepted. Anticoagulation was deliberately targeted toward the lower end of the usual range and never exceeded 280 seconds, limiting peripartum bleeding risk while preserving protection against transseptal thrombus formation; the same consideration led the anesthetic team to select intravenous rather than neuraxial analgesia. Fluoroscopy was limited to 13 minutes, an exposure at which the fetal dose arising from internal scatter at term remains well below the thresholds associated with deterministic effects. Mean left atrial pressure, although substantially reduced, remained at 26 mm Hg immediately after dilation, and pulmonary artery systolic pressure had regressed only partially by the first postpartum day. Relief of the obstruction is therefore immediate, but reversal of pulmonary vascular changes is not, which is why the first 24 postpartum hours were spent under continuous monitoring in the coronary care unit rather than on a conventional ward.

The decision was individualized. Features favoring intervention were class IV dyspnea, a valve area of 0.55 cm², a mean gradient of 17 mm Hg despite a resting heart rate below 70 beats/min, pulmonary artery systolic pressure of 58 mm Hg, and the expected hemodynamic burden of advancing labor and postpartum autotransfusion. Features supporting feasibility were sinus rhythm, preserved systemic pressure, absence of clinical congestion, no visible left atrial thrombus, and a valve morphology still amenable to balloon commissurotomy, the borderline Wilkins score of 9 being attributable mainly to subvalvular thickening with only moderate leaflet calcification and preserved mobility. The early stage of labor created time for catheter treatment while avoiding the delay and physiological burden of cardiac surgery. The increase in valve area to 1.60 cm², reduction in transmitral gradient, minimal regurgitation, and uncomplicated spontaneous vaginal delivery support the success of this strategy in this patient.

Several limitations must be acknowledged. The total radiation dose, estimated blood loss, and birth weight were not recorded in the available clinical documentation, nor was the duration of labor before admission. The absence of long-term maternal and neonatal follow-up also limits conclusions. This single case should not be interpreted as evidence that catheter intervention during labor is routinely preferable. It instead illustrates how rapid multidisciplinary assessment may identify a narrow window in which relief of a critical obstruction can precede delivery.

Follow-up

No complication occurred during the procedure, delivery, or the 24-hour period of coronary care unit monitoring. The newborn was delivered at term with Apgar scores of 8 at one minute and 10 at five minutes and had an uncomplicated immediate course. Longer-term maternal and neonatal follow-up was not available.

## Conclusions

Critical rheumatic mitral stenosis that remains untreated when a patient presents at term in early labor creates a complex sequencing problem. In this patient, urgent Inoue-balloon commissurotomy performed approximately nine hours before spontaneous vaginal delivery increased mitral valve area to 1.60 cm² and reduced the transmitral gradient without significant mitral regurgitation or immediate maternal, obstetric, neonatal, or procedural complications. Because long-term maternal and neonatal follow-up was not available, these findings should be interpreted as immediate in-hospital outcomes only. The case emphasizes individualized Pregnancy Heart Team decision-making rather than a universal management pathway.
